# An efficient, free-breathing protocol for MR right heart catheterization

**DOI:** 10.1186/1532-429X-16-S1-T5

**Published:** 2014-01-16

**Authors:** Jonathan R Mazal, Toby Rogers, Anthony Z Faranesh, Peter Kellman, William Schenke, Annette Stine, Laurie Grant, Kanishka Ratnayaka, Robert J Lederman

**Affiliations:** 1 National, Heart, Lung, and Blood Institute - NIH, Bethesda, Maryland, USA; 2 Children's National Medical Center, Washington DC, District of Columbia, USA

## Background

MR right heart catheterization is a valuable diagnostic tool in patients with cardiac and pulmonary vascular disease. Many patients are dyspneic and/or require moderate sedation so MR examinations with multiple breath holds are not feasible. We describe an efficient free-breathing MR imaging protocol designed to facilitate MR catheterization in these challenging patients.

## Methods

The baseline MR examination begins with cardiac localizers. At this stage it is important to verify isocenter because if the patient is too deep in the bore then femoral vascular access sheaths are inaccessible to the operator. Through-plane phase contrast (PC) scans in the ascending aorta and main pulmonary artery follow for quantification of Qp and Qs. To avoid the need to repeat long PC scans (3 averages), we first perform brief PC 'localizer' scans with a single average to confirm the selected velocity-encoding factor is correct. For cardiac volumetric analysis, long and short axis views are obtained using a real-time SSFP sequence that sacrifices some spatial resolution for increased temporal resolution. Data is collected over one and a half heartbeats to ensure the entire cardiac cycle is captured. Because of the delay in image reconstruction, the short axis stack is acquired last while the operator prepares for catheterization. A dedicated user interface (Interactive Front End, Siemens; Erlangen, Germany) is used for real-time imaging during catheterization. This interface permits interactive manipulation of imaging planes, slice thickness, saturation pre-pulse, and cine loop recordings. To minimize catheterization time, reference images consisting of orthogonal views of the superior and inferior vena cavae, the right ventricular outflow tract, and the main and branch pulmonary arteries are prescribed prior to commencing catheterization. If additional physiological provocations during catheterization are indicated, then the baseline imaging sequences are repeated during each provocation via automatic propagation of the sequence parameters.

## Results

Acquisition order was optimized to shorten scan times by enhancing scanner duty cycle. With practice we reduced the baseline MR examination time from 50 minutes to 30 minutes. With parameter duplication, subsequent scan time during physiological provocations was reduced to 8 minutes per provocation. We are now able to perform baseline MR examination, right heart catheterization and multiple physiological provocations.

## Conclusions

This free-breathing MR examination protocol enables MR catheterization with multiple physiological provocations to be performed in patients with dyspnea and/or under moderate conscious sedation.

## Funding

This work is supported by the Division of Intramural Research (Z01-HL005062-08, Z01-HL006039-01), National Heart Lung and Blood Institute, National Institutes of Health, USA.

**Table 1:** MR Right Heart Catheterization Pulse Sequence Parameters

	Tradional Localizers	Multi-Slice Localizer	Localizer Phase Contrast	Diagnostic Phase Contrast	Real-Time LAX Cine	Real-Time SAX Cine	Real-Time SSFP (IFE)
TR	2.66 ms	2.81 ms	4.64 ms	4.84 ms	2.58 ms	2.46 ms	2.44 ms

TE	1.13 ms	1.23 ms	2.47 ms	2.64 ms	1.13 ms	1.04 ms	1.22 ms

Matrix (Readout × Phase)	168 × 256	160 × 240	173 × 192	256 × 256	80 × 160	80 × 160	128 × 128

Slice Thickness	8.0 mm	4.5 mm	6.0 mm	6.0 mm	6.0 mm	8.0 mm	8.0 mm

Flip Angle	80°	80°	20°	20°	45°	45°	45°

Acceleration Factor	2	2	2	2	4	4	4

Cardiac Gating	None	None	ECG/Retro	ECG/Retro	ECG/Trigger (1.5 Beats)	ECG/Trigger (1.5 Beats)	None

Scan Times	10 sec	35 sec	23 sec	2-3 min each	3 sec	35 sec	Infinite Measurements

**Figure 1:**
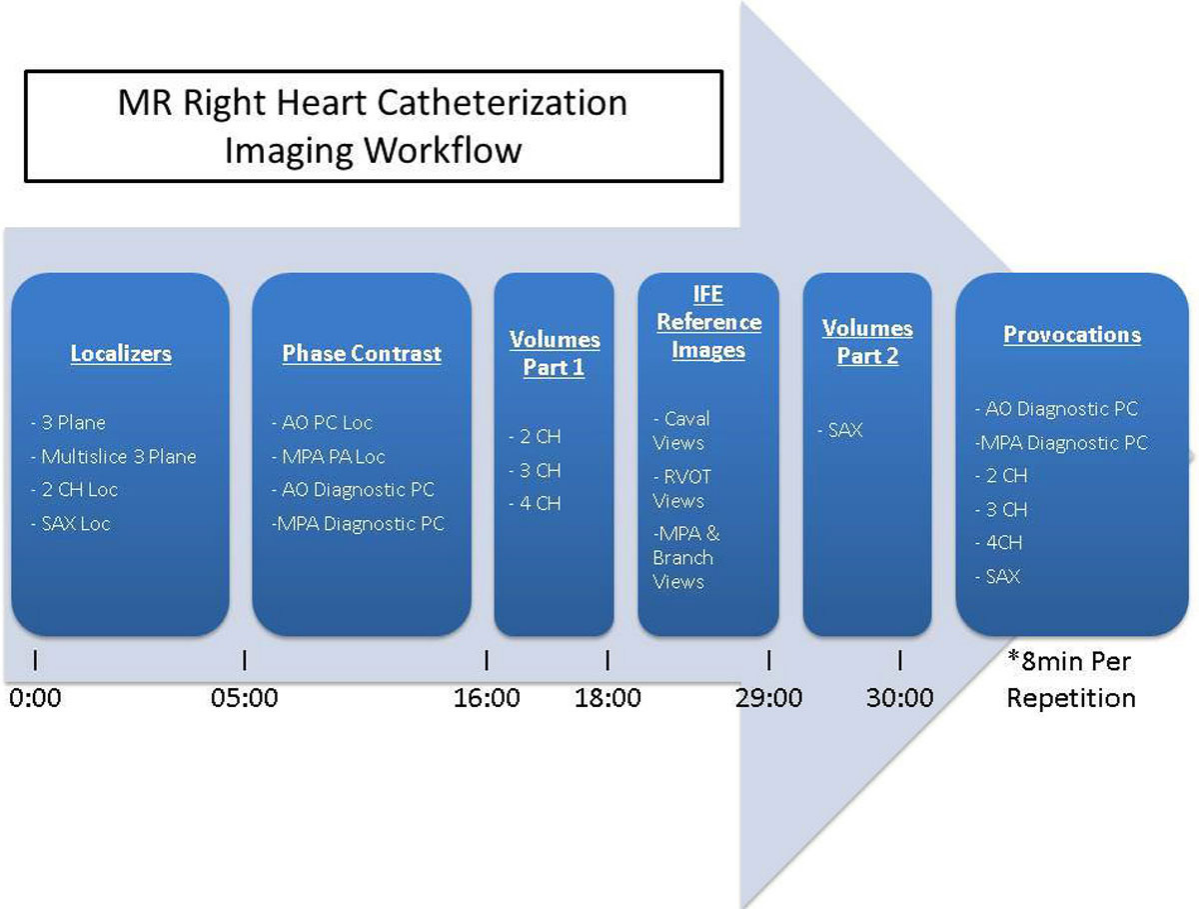
**Diagram of pulse sequence workflow for MR right heart catheterization, including time markers indicating start of exam, duration of imaging sections, and total time at completion of baseline imaging, prior to start of catheterization procedure**. Total time of repeat imaging asociated with procedural provocations depends on prognostic value as determined by primary operator.

